# Five-factor model, technology enthusiasm and technology anxiety

**DOI:** 10.1177/20552076231203602

**Published:** 2023-09-20

**Authors:** Jessica Berner, Ana Luiza Dallora, Bruna Palm, Johan Sanmartin Berglund, Peter Anderberg

**Affiliations:** 1 Department of Health, 4206 Blekinge Institute of Technology, Karlskrona, Sweden; 2 Department of Mathematics, 4206 Blekinge Institute of Technology, Karlskrona, Sweden; 3 School of Health Sciences, University of Skövde, Skövde, Sweden

**Keywords:** Technology anxiety, technology enthusiasm, older adults, personality, five-factor model, digital social participation

## Abstract

Older adults need to participate in the digital society, as societal and personal changes and what they do with the remaining time that they have in their older years has an undeniable effect on motivation, cognition and emotion. Changes in personality traits were investigated in older adults over the period 2019–2021. Technology enthusiasm and technology anxiety are attitudes that affect the relationship to the technology used. The changes in the score of technology enthusiasm and technology anxiety were the dependent variables. They were investigated with personality traits, age, gender, education, whether someone lives alone, cognitive function, digital social participation (DSP) and health literacy as predictors of the outcome. The Edwards-Nunnally index and logistic regression were used. The results indicated that DSP, lower age, lower neuroticism and higher education were indicative of less technology anxiety. High DSP and high extraversion are indicative of technology enthusiasm. DSP and attitude towards technology seem to be key in getting older adults to stay active online.

## Introduction

Time is an integral part of all psychological phenomena.^1^^(p.1913)^

Time has been linked positively with personality trait change. Previously it was considered that personality traits were obtained in young adulthood and then remained stable throughout a lifetime. Traits developed, they were structured and tested until midlife and then they stabilised.^2^

However, it appears that personality development and change, measured by the mean level of changes in traits, are more frequent than expected, even until the age of 80+.^3^ Previous research has agreed that if a personality trait is changed at a point in time during adulthood, it will remain changed.^4^ Also, changes in personality traits are noted normally within a positive context. However, with mild cognitive impairment, and early stages of dementia negative personality change is common. For those with beginning dementia, often negative emotions affect a person's quality of life as well as the caregivers.^5^ Suffering from chronic illnesses, can also alter how an older person reacts and appreciates in daily living.

Personality has been linked to acceptance, usage and enthusiasm for technology in older adults. Engaging positively contributes a lot to technology enthusiasm/acceptance, and a positive feeling in daily living. Technology use also plays a role in personality development in ageing.^6^

The more frequent exposure and use of technology tends to decline anxiety and its novelty; specifically with the use of the internet it has been shown that it helps maintain cognitive function,^7^ which in turn affects personality.

There are many older users today, which are connected to the three levels of the digital divide. The first level refers to access to and rapidity; the second level has to do with skills and use, and the third level is the outcomes of the usage.^8,9^ Older adults who are now online usually fall into at least one of the three divides.

The outcome of usage is directly linked to ageing well. This is determined by the ability to remain mentally and physically well and has the potential to keep healthy engagement in life,^10^ as well as a positive influence on maintaining good cognitive function. So being (active) online, having social relations and social connectedness play a role. Motivational aspects of what the older adult does online may need to be considered. The deepening of understanding and expertise in already satisfying areas of life will help technology use. On the contrary, the negative stance on how the internet may not be safe, or the anything goes wrong with spyware, for example, may block the older person.

Older adults’ attitudes towards technology use have much to do with the regions and countries they live in. The attitudes towards internet use begins with the frequency and ease of use. For example in 2019 that there was a significant difference in older adults who started using the internet (11.6%) compared with those who stopped (3.1%).^7^ Swedish older adults display a positive attitude towards information communication technology (ICT) devices, as well as more frequent usage of the internet.^11^ More recent research from Spain has indicated that older adults value ICT yet compared with younger adults, they may need different interfaces and technical help,^12^ which makes them have an open attitude towards new technology.

Changes with age affect interactions with technology, but can also in turn design better products for older adults.^13^ Attitudes towards technology use are of importance in so far that it gives insight into how the technology is adopted. With active ageing/ageing in place, many new technologies in homes are known to be influenced by age, privacy, gender, income, living with someone, and geographic location are all factors which impact attitude towards technology and affect usage.^14^

## Research question/aim of the paper

Technology enthusiasm and technology anxiety are attitudes that affect the relationship to the technology used and can influence continuity and any new adoption of an etechnology.

This research investigated personality traits, age, gender, education, whether someone lives alone, cognitive function, digital social participation (DSP) and health literacy as predictors on the outcome change in score of technology enthusiasm and technology anxiety from 2019 to 2021.

**RQ1:** Are there changes in personality traits of older adults in SNAC-Blekinge over time?
**RQ2:** Do these personality traits affect change in score of technology anxiety and enthusiasm?
**RQ3:** Over time, which variables affect technology enthusiasm and technology anxiety: age, gender, education, living alone/not, cognitive function, digital social participation and health literacy?

## Measures

### Personality traits

The Big Five or Five-Factor model (FFM) contains five personality traits: neuroticism (N), openness (O), extraversion (E), conscientiousness (C) and agreeableness (A). These are built on specific definers with Likert scales and trait adjectives and are composed of 60 items in a questionnaire.^15^ The traits work separately and can increase or decrease in intensity throughout a life span.

One major advantage of the FFM is that older adults have been used in the development and validation of the instrument.^15^

### Digital social participation

DSP is considered an outcome. It is a short instrument for older adults 65 years and above measuring the perception of the benefits of their online social participation (excluding specific uses of applications). It is a reduced questionnaire from 10 to six questions regarding social connectedness and social relations.^16^ It verifies how much this relationship between social participation and well-being is connected with the internet. It is also there irrespective of specific services and applications.

### Technology enthusiasm and anxiety

Technology acceptance by older adults (Technology PH) was measured through two factors, either technology anxiety (techAnxiety) or technology enthusiasm (techEnthusiasm).

There are six questions, three answering one factor and three answering the other. Technology PH is assessed independently, techAnxiety and techEnthusiasm also. Partly to verify whether the score contributes to one total measure (techPh) and two separate measures – anxiety and enthusiasm as separate outcomes.

### Internet use

Internet use has evolved where people establish an online behaviour (which is in accordance with certain personality traits). Many have a working opinion on the information on the internet. For example, day-to-day connections to reading the news, playing online card games such as Bridge, or setting up and using profiles (such as online dating).

Internet use and a sense of community with older adults have shown to be quite strong. This combined with conscientiousness, agreeableness and openness have previously been strong predictors in adoption of internet use. Certain traits such as neuroticism and extraversion have typically been associated with certain types of internet behaviour, such as addiction to smartphones. This data, however, stops at the age of 68.^17^

Being less lonely was a difference with Internet users; for example, older adults who scored low on social capital in general had higher levels of social capital compared with those who did not use the internet.^18^

It can be assumed that personality characteristics contribute both to positive and negative outcomes with regard to technology enthusiasm and technology anxiety. Agreeableness, conscientiousness and extraversion are inversely related to more traditional uses of the internet,^19^ such as emailing chatting and browsing online.^20^

## Method

For the statistical analyses, SPSS 28.01.01 was used.

The study originated from the Swedish National Study on Aging and Care (SNAC). This is a longitudinal study investigating ageing over time, and began in 2001.^21^ The cohort contains people aged 60 years and above in the following age groups: 60, 66, 72, 78, 81, 84, 87, 90, 93 and 96+ years. The data covers physical assessments, psychological and cognitive function and technology use among others. The SNAC data is collected from four sites, namely Skåne, Kungsholmen, Nordanstig and Blekinge.

**Figure 1.**
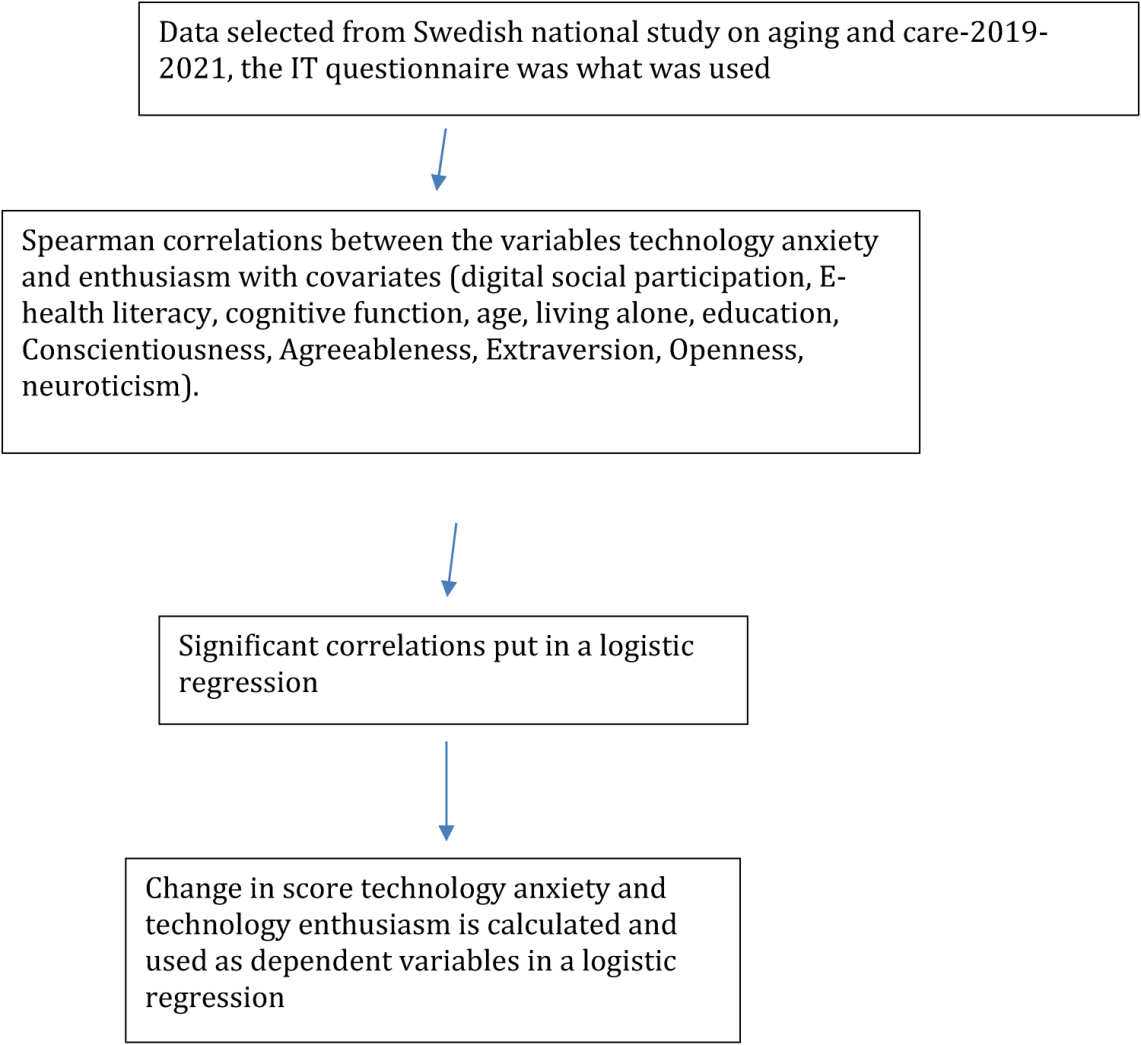
Different phases of the study.

The sample in this study is from SNAC specifically from the Blekinge region. The authors used data collected from the longitudinal study from two different times 2019 and 2021. The reason for this selection has to do with the frequency of the IT questionnaire that is given in SNAC. The older adults (*N*  =  393, *M*_age_  =  78.2 years) completed personality and technology use questionnaires, in addition to a self-report measure of perceived general health. Correlational analyses were performed to examine the basic relations between these constructs (please see Figure 1).

### Statistical procedures

First, the authors ran a Shapiro-Wilks test, which indicated strong evidence against the assumption of normality. Non-parametric tests were used after that namely, Spearman rho to see the correlations between variables.

In order to investigate the changes of personality over time, the authors looked at the mean score between T2 and T1 for the personality traits to observe whether there were any major changes.

To work with the change in score between the years T1 (2019) and T2 (2021), we used the outcome variable technology anxiety and technology enthusiasm and used the Edwards-Nunally index. This calculates the change in Tech PH over time. The following formula was applied: XT2 < (Cronbach's alpha × (Xt1 – mean)  +  mean – 1.960 × standard error). It calculates change in score of technology anxiety and technology enthusiasm of the older adult between 2019 and 2021 and avoids the regression to the mean.

If the change in score is significant it will be seen through the logistic regression with the selected covariates that affect Tech PH. The score is either a decline or no decline (decline being negative with regard to technology enthusiasm) and no decline being negative for technology anxiety as that means it is on the increase.

For the logistic regression, a Cox Snelle Square (*R*^2^: 0.2–0.4) indicates a good model.

Cronbach's alpha was, respectively, for TechAnxiety (0.583) and Tech Enthusiasm (0.779).

## Results

Table 1 describes the sample characteristics for Swedish Older adults living in Blekinge in time T1 (2019) and T2 (2021).

**Table 1.** Distribution of the baseline characteristics.

*N* = 385	T1	T2
Age range	63–99	66–101
Age mean	75.21	77.44
Female	201 (52.2%)	201 (52.2%)
Male	184 (47.8%)	184 (47.8%)
Education lower N360	121 (33.6%)	121 (33.6%)
Education middle	119 (33.1%)	119 (33.1%)
Education high	120 (33.3%)	120 (33.3%)
Live alone	109 (28.3%)	171 (35%)
Live with someone	272 (70.6%)	318 (65%)
Neuroticism	25.4	24.97
Openness	37.86	37.34
Extraversion	40.78	40.05
Agreeableness	46.48	**46.96**
Conscientiousness	47	46.29
Digital social participation (DSP) mean	3.08	**3**.**13**
Health literacy score mean	3.06	**3**.**42**
Cognitive function mean	28.95	28.27
Tech PH	3.16	2.8
Use the internet		

TechPH: Technology anxiety and enthusiasm scoring high is more on either scale.

DSP, Eheals: the score is Likert scale 1–5 (5: totally agree) OCEAN personality traits: scoring higher is more of each variable. The % is the percentage of the total *N*. The bold values indicate an increase in score with time.

From here we investigated the correlations between the variables, technology anxiety and technology enthusiasm with all the covariates: DSP, Ehealth literacy (Eheals), cognitive function, age, living alone/not, education level, conscientiousness, agreeableness, extraversion, openness, and neuroticism.

The significant variables from the Spearman correlations were included in a backward logistic regression, with change in score as the dependent variable between 2019 and 2021 of Technology PH (Table 2).

**Table 2.** Spearman correlations in *R*.

TECH ENTHuSIASM	Spearman rho	Significance
Digital social participation (DSP)	0.508	*p* = 0.001
eHeals	0.489	*p* = 0.001
Mini mental state examination (MMSE)	0.194	*p* < 0.001
Education level	0.176	*p* < 0.001
Age	(−0.276)	*p* < 0.001
Conscientiousness	0.309	*p* < 0.001
Agreeableness	0.0849	NS
Openness	0.136	*p* < 0.05
Extraversion	0.225	*p* < 0.001
Neuroticism	(−0.268)	*p* < 0.001
Live alone with someone	(−0.068)	NS

TECH Anxiety	Spearman rho	Significance
Digital social participation (DSP)	(−0.0138)	*p* = 0.001
eHeals	(−0.25)	*p* = 0.001
MMSE	(−0.146)	*p* < 0.001
Education level	(−0.155)	*p* < 0.001
Age	0.244	*p* < 0.001
Conscientiousness	(−0.274)	*p* < 0.001
Agreeableness	(−0.22)	*p* < 0.001
Openness	(−0.025)	NS
Extraversion	(−0.217)	*p* < 0.001
Neuroticism	−0.356	*p* < 0.001
Live alone with someone	(−0.221)	*p* < 0.001

The Logistic regression models are shown in Tables 3 and 4: with technology anxiety, the people who score higher on neuroticism are the ones who are significantly more anxious with regard to technology adoption and use. DSP is also a factor where the less an older person participates digitally the more anxious he/she is. Lower education and higher age are also characteristics, which affect technology anxiety.

**Table 3.** Logistic regression with change in score attitude towards technology (cox snelle 0.148).

Final model after backward regression				
**Anxiety** (*N* = 215)	Coeff	Odds ratio	95%	Significance
Digital social participation (DSP)	(−0.371)	0.69	0.519–0.917	*p* < 0.05
Age	0.081	1.085	1.030–1.142	*p* < 0.01
Neuroticism	0.049	1.05	1.005–1.098	0.05
Education	(−0.457)	0.633	0.424–0.945	0.05

**Table 4.** Logistic regression with change in score attitude towards technology (cox snelle 0.140).

Final model after backward regression				
**Enthusiasm** (*N* = 188)	Coeff	Odds ratio	95%	Significance
Digital social participation (DSP)	(−0537)	0.584	0.427–0.800	*p* < 0.001
Extraversion	(−0.083)	0.92	0.873–0.970	*p* < 0.01

Extraversion is indicative of technology enthusiasm. Scoring high on extraversion has a negative relationship with a significant change in score in technology enthusiasm. DSP also has a negative relationship with technology enthusiasm going down over time.

## Discussion

The personality traits in this study were quite stable throughout the three years, with only small declines in intensity (which are normal). In this study, the cognitive score also did not change so much throughout the years, which is a positive finding with regard to cognitive decline. The mean was 28.95 in 2019 and 28. 27 in 2021. Extraversion and neuroticism indicated significant changes in technology enthusiasm and technology anxiety scores. Previous studies on smartphone addiction showed that neuroticism was strongly correlated with smartphone addiction where people tended to control their relationships compulsively by checking their social media and messaging. Extraversion was a way to fulfil social interactions.^17^

In older adults, internet non-users have been found to score lower on extraversion and higher on neuroticism.^22^ Behavioural changes and responses to how to participate in the digital society are part of psychological phenomena.

Scoring high on DSP is indicative of being online a significant amount and also feeling comfortable using etechnologies, and ehealth applications. The higher the age the less DSP; however, the mean score of DSP was on the increase, from 3.03 to 3.13 in 2021. Technology acceptance and learning new technology influence technology enthusiasm.^23^ DSP is strongly influenced by age.^24^ It is an instrument that also has indicated that those feeling lonely do not tend to go online/or do not have the social online element.

Those higher in age and lower educated were significantly higher on their scores of technology anxiety.

Eheals was also on the increase mean in 2019 (3.06) and in 2021 (3.42). E-health literacy is important as it is a solution to the healthcare costs that are rising and the lack of workforce for older adults needing care at home/and in healthcare. It is on the increase in this study; we should take into consideration the COVID-19 pandemic, which influenced being online more. But also, in general, ehealth is welcome positively by older adults. It is believed to improve the quality of care.^23^

## Conclusion

In conclusion, technology anxiety is on the increase and technology enthusiasm is on the decrease over time. Age-related motivational changes have been shown to lead older adults to process emotionally meaningful goals (both good and bad) so as to maintain an emotional equilibrium.^25^

A recent study on psychological adjustment into adulthood indicated that ICT was not affecting these technology enthusiasms or anxieties that much.^6^ Furthermore, anxiety has often been more prevalent in younger adults than older adults.^26^ Prioritising emotion regulation as you get older, through cognitive processing indicates that emotion regulation remains quite intact, where the personality traits are individualistic and age-independent.

What is clear in this study is participating online and remaining active online is important. It may be important to provide better support for the elderly in their older years, with better DSP and a positive attitude towards technology (Technophilia), this can turn into a quality in an individual's relationship towards the technology.^16^ A reliance on feelings instead of memory for details, can be an option and lead to better decision quality in older adults.^25^

## Limitations

The question about peer support with regard to technology seems to be something that is key to help with understanding the attitude towards technology, and how optimistic a person stays towards new development in the field. It may be that thinking that you cannot use technology is just a way of thinking, but in actual fact with the support and tools around it is better than expected. Usually, technology anxiety indicates a lack of ability to handle technology on their own and a fear of social isolation and a lack of support for the ageing population.^16^ Technology enthusiasm may be overestimated; perhaps it is great just as long as I do not have to use it myself. The older adults may be just wanting to impress the nurses by giving the questionnaire (experimenter bias).

There is most likely a presence of a historical effect, the older adults are triggered to use technology during the COVID-19 pandemic, which increases their enthusiasm for it.
